# The Prevalence of Endometriosis in Patients with Unexplained Infertility

**DOI:** 10.3390/jcm13020444

**Published:** 2024-01-13

**Authors:** Camran Nezhat, Farrah Khoyloo, Angie Tsuei, Ellie Armani, Barbara Page, Thomas Rduch, Ceana Nezhat

**Affiliations:** 1 Stanford University Medical Center, Palo Alto, CA 94305, USA; 2 University of California San Francisco, San Francisco, CA 94143, USA; 3 Camran Nezhat Institute, Center for Special Minimally Invasive and Robotic Surgery, Woodside, CA 94061, USA; 4 University of California Berkeley, Berkeley, CA 94720, USA; 5 Laboratory for Particles Biology Interactions, Swiss Federal Laboratories for Materials Science and Technology (Empa), CH-9014 St. Gallen, Switzerland; 6 Department of Gynecology and Obstetrics, Cantonal Hospital St. Gallen (KSSG), CH-9007 St. Gallen, Switzerland; 7 Nezhat Medical Center, Atlanta Center for Special Minimally Invasive Surgery and Reproductive Medicine, Atlanta, GA 30342, USA

**Keywords:** endometriosis, unexplained infertility, prevalence, endometriosis and cancer, endometriosis and ovarian hyperstimulation, pregnancy complications

## Abstract

Endometriosis, a systemic ailment, profoundly affects various aspects of life, often eluding detection for over a decade. This leads to enduring issues such as chronic pain, infertility, emotional strain, and potential organ dysfunction. The prolonged absence of diagnosis can contribute to unexplained obstetric challenges and fertility issues, necessitating costly and emotionally taxing treatments. While biopsy remains the gold standard for diagnosis, emerging noninvasive screening methods are gaining prominence. These tests can indicate endometriosis in cases of unexplained infertility, offering valuable insights to patients and physicians managing both obstetric and non-obstetric conditions. In a retrospective cross-sectional study involving 215 patients aged 25 to 45 with unexplained infertility, diagnostic laparoscopy was performed after unsuccessful reproductive technology attempts. Pathology results revealed tissue abnormalities in 98.6% of patients, with 90.7% showing endometriosis, confirmed by the presence of endometrial-like glands and stroma. The study underscores the potential role of endometriosis in unexplained infertility cases. Although the study acknowledges selection bias, a higher than previously reported prevalence suggests evaluating endometriosis in patients who have not responded to previous reproductive interventions may be justified. Early detection holds significance due to associations with ovarian cancer, prolonged fertility drug use, pregnancy complications, and elevated post-delivery stroke risk.

## 1. Introduction

Endometriosis is a chronic, inflammatory, systemic, and estrogenic-dependent disease [1,2]. This condition may manifest genitally or extragenitally [1,3,4,5]. The prevalence of endometriosis ranges up to over a billion individuals globally [6,7,8,9,10,11,12]. A combination of laparoscopic and hysteroscopic techniques is used to detect endometriosis and other potential intrauterine and intra-abdominal abnormalities [13]. This condition impacts a significant number of patients, encompassing up to 80% of those who experience pelvic pain [6]. Moreover, endometriosis affects 47% of patients seeking gynecological care [9]. Endometriosis patients also have a higher rate of obstetrical complications [14]. Endometriosis is present in up to 87% of patients with symptomatic uterine leiomyomas [8,11]. In addition, endometriosis has the potential for malignant transformations [10,15,16]. It is estimated that more than a billion women and gender-inclusive individuals worldwide will be affected by endometriosis during their reproductive years [8,11,12]. Endometriosis is also closely related to cardiovascular disease, hypertension, and dyslipidemia [17,18]. Patients with endometriosis are more likely to develop ischemic heart disease (40%) and cerebrovascular disease (19%) [19]. Other conditions that are more common in patients with endometriosis compared to the general US population include: hypothyroidism, fibromyalgia, chronic fatigue syndrome, autoimmune diseases, allergies, and asthma [20]. There is also some evidence of an association between endometriosis and autoimmune diseases such as systemic lupus erythematosus, Sjogren’s Syndrome, rheumatoid arthritis, celiac disease, multiple sclerosis, and inflammatory bowel disease [21].

Due to potential stigma and barriers to healthcare access, many cases go undiagnosed for approximately 11 years [22,23]. As a result, individuals silently endure the physical and emotional burdens of endometriosis, including chronic pain, dysmenorrhea, dyspareunia, dysuria, abnormal urinary and bowel habits, infertility, and organ dysfunction, without finding relief [3,4,5]. The impact of endometriosis extends to multiple facets of life, such as obstetrical complications, silent loss of organ function and increased risk of ovarian cancer, strained relationships, heightened depression and anxiety, financial difficulties arising from costly fertility treatments, and absence from work [13,24,25,26,27,28,29]. For those who experience “unexplained infertility,” the absence of a diagnosis can lead to an emotionally taxing journey and, ultimately, a decline in health-related quality of life [30,31].

Infertility refers to the inability to conceive after 12 or more cycles of unprotected intercourse for individuals under thirty-five years old or after six cycles for those over thirty-five years old [32]. The leading causes of infertility are ovulatory dysfunction, tubal disease, and male-factor infertility [33]. However, in cases of unexplained infertility, standard investigations, including tests for ovulation, tubal patency, and semen analysis, yield normal results [34]. Thus, the criteria for unexplained infertility include couples that have normal findings to the aforementioned tests. It has been reported that approximately 30% of couples facing infertility issues are diagnosed with unexplained infertility [32]. These individuals may undergo years of treatments, such as hormonal treatments, intrauterine insemination (IUI), and in vitro fertilization (IVF), without receiving an explanation for their unsuccessful outcomes. An 18-month prospective cohort study revealed that the median costs per individual for infertility treatments exhibit a spectrum, spanning from USD 1182 for hormonal medications to a substantial USD 38,015 for IVF with or without donor eggs. The calculated expense associated with attaining a successful pregnancy via IVF was estimated at USD 61,377 [35]. Many infertility therapies may involve ovarian hyperstimulation, which can result in elevated estrogen levels and the exacerbation of endometriosis symptoms.

When evaluating infertility patients, the recommendation against laparoscopy as a routine procedure has generated considerable debate among stakeholders [36,37]. During the evaluation of unexplained infertility, pelvic ultrasound is often obtained to rule out anatomic pathology. Based on a 2016 Cochrane Review, no evaluated imaging modalities, including transvaginal ultrasonography (TVUS) and magnetic resonance imaging (MRI), were able to detect endometriosis in the pelvis with enough accuracy to replace surgery [38]. For endometriomas specifically, transvaginal ultrasound has a 93% sensitivity and 96% specificity [38]. MRI is a second-line imaging modality. For endometriomas, MRI has a high sensitivity (95%) and specificity (91%) and better sensitivity for detecting co-existing deep infiltrating endometriosis [38]. Current evidence-based guidelines recommend against the use of routine diagnostic laparoscopy for the diagnosis of unexplained infertility, as they state that there is insufficient good-quality data to suggest that clinically relevant diagnoses will be missed by omitting a laparoscopy [36]. The current gold standard for diagnosing endometriosis is laparoscopic surgery or laparotomy with histological examination to validate any clinical suspicion that arose during the laparoscopic visualization [22,36,38,39]. In cases where an incidental finding of endometriosis has been identified at laparoscopy, the 2023 ESRE guidelines recommend that unexplained infertility no longer be considered as the diagnosis. However, if physicians were able to screen for or diagnose endometriosis with high accuracy non-invasively, it may affect their patients’ management. Non-invasive methods, including but not limited to saliva testing, endometrial function testing, the Nezhat Endometriosis Risk Advisor (EndoRA) mobile application, and BCL6 testing, can be highly suggestive of endometriosis [40,41,42,43]. Other non-invasive diagnosis methods are also currently being researched, such as the use of transforming growth factor-β-induced protein ig-h3 (TGFBI) as a biomarker for endometriosis, or using Fourier transform infrared vibrational spectroscopy to study the association between protein and lipid fraction and endometrioma volume [44,45].

According to medical literature, the current prevalence of endometriosis in individuals with unexplained infertility is reportedly 30–63.2%, while the occurrence of abnormal pathological discoveries is documented to reach up to 80.7% [46,47,48,49,50,51,52]. Furthermore, histologic diagnosis has long been the gold standard of diagnosis for endometriosis. Visual diagnosis has a positive predictive value of 45%, sensitivity of 97%, with negative predictive value of 99%, and specificity of 77% [53]. A recent study also reported a low positive predictive value of visual diagnosis laparoscopically of 39% [54]. This may in part be due to diagnostic issues of both stromal and glandular components [55]. For stromal components, alterations in the typical microscopy can make detection difficult. In some cases, endometriotic stroma can be very subtle and limited to a barely noticeable and frequently discontinuous zone that is around the gland or oppositive the epithelial lining of an endometriotic cyst. For these instances, CD10 immunostaining may be helpful. For the glandular component, detection may be made difficult by medication effects or metaplastic changes. In some rare cases, atypia may alter histologic appearance and is in part how cancer may arise from endometriosis lesions and cysts [55]. Such a case of endometroid adenocarcinoma arising from endometriosis has been presented and demonstrates the potential for cellular atypia in endometriosis lesions [56]. Additionally, in cases of endometriosis, especially minimal or Stage I disease, there is a potential that lesions could unintentionally be compromised during excision, posing a challenge to achieving a thorough histological analysis. Another limitation is that the pathologist may miss the endometriosis by examining the tissue not involved in endometriosis during sampling.

The existing definition of endometriosis relies on identifying endometrial-like epithelial and stromal cells at atypical locations within the body. However, emerging insights indicate that certain disease variants lack endometrial-like epithelial and stromal cells [57]. In such cases, the presence of hemosiderin-laden macrophages alone indicates endometriosis [58,59,60]. In addition, a consistent presence of smooth muscle and fibrous tissue is evident across many different types of endometriosis [57,61]. Consequently, Vigano et al. have proposed a new definition for endometriosis as “a fibrotic condition characterized by the presence of identifiable endometrial stroma and epithelium” [57]. The primary objective and purpose of this study is to assess the true prevalence and intricate severity of endometriosis in a distinctive cohort of patients who are facing unexplained infertility and have failed prior infertility treatment.

## 2. Materials and Methods

This is a retrospective cross-sectional study from September 2019 to March 2023. The study was approved by the West Coast IRB committee.

### 2.1. Subjects and Data Collection

For data collection in this study, an electronic health record system (Practice Fusion, San Francisco, CA, USA) was used. Information was extracted from various sources within each patient’s medical chart, including the patient intake health questionnaire, operative report, and pathology report. All patients underwent a comprehensive evaluation and treatment by their reproductive endocrinologists (REIs) to investigate the reasons behind failed infertility treatments and unexplained infertility. Prior to seeking consultation for the diagnostic laparoscopy and potential endometriosis treatment, patients had undergone a variety of unsuccessful infertility treatments. These treatments included assisted reproductive technology (ART) such as hormonal medications, intrauterine insemination (IUI), in vitro fertilization (IVF), or a combination thereof. Subsequently, these patients were referred for minimally invasive surgery (MIS) for surgical diagnosis and possible treatment for any possible pathology.

**Figure 1:**
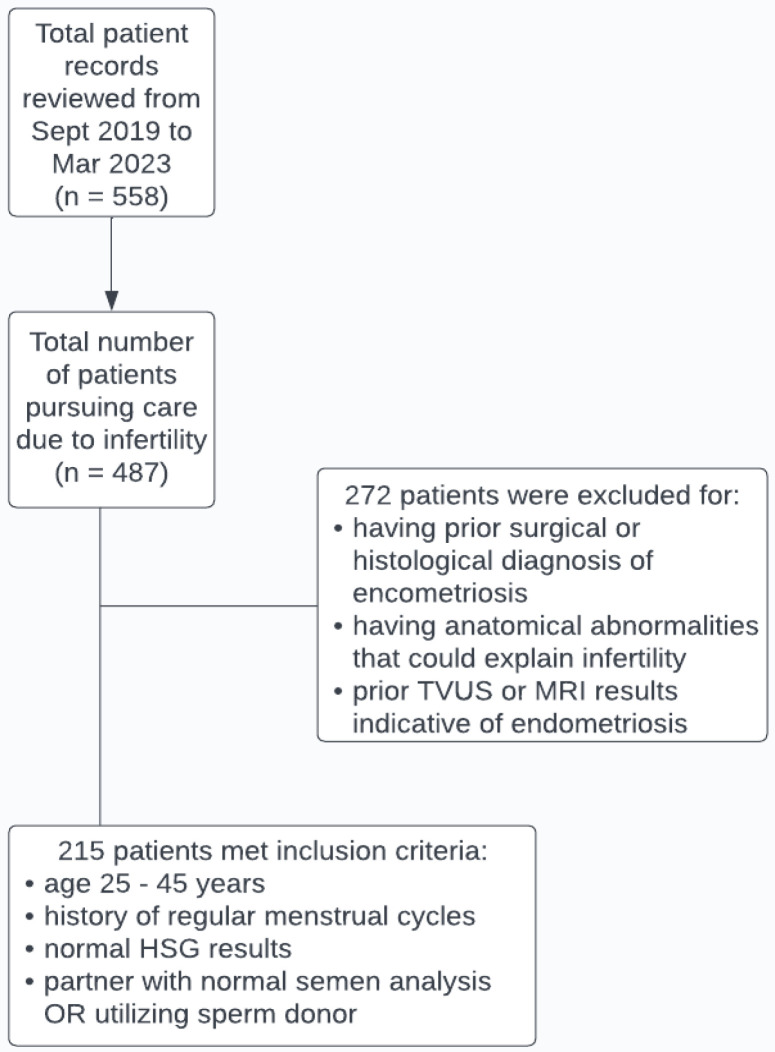
This flow chart represents a summary of the examination that was performed when determining which patients were eligible for this study.

A comprehensive examination was conducted, involving a total of 558 patient records. Among these patients, 487 were discerned as having pursued medical care due to infertility. The inclusion criteria for cases of unexplained infertility involved individuals aged 25 to 45 years, with this age range being set based on the youngest and oldest patients who presented a concern for infertility at the consultation. Inclusion criteria also involved possessing a history of regular menstrual cycles (defined as lasting 28 to 35 days), displaying normal HSG results, and either having a partner with a normal semen analysis or opting to utilize a sperm donor. These additional inclusion criteria were imposed to identify patients with unexplained infertility. The exclusion criteria included having a prior surgical or histological diagnosis of endometriosis and other confounding variables, such as anatomical abnormalities that could explain infertility. A prior transvaginal ultrasound and MRI results indicative of endometriosis were also considered when excluding patients. The inclusion and exclusion criteria were used as a guideline when further analyzing patient records to determine which of the 487 patients presenting a concern for infertility had unexplained infertility and would be qualified to be a study participant. Figure 1 represents the 215 patients that ultimately met the criteria and were included in the study. 

### 2.2. Surgical and Histological Evaluation

Having been presented with the option to consider alternative treatments for unexplained infertility, such as GnRH agonists or antagonists, aromatase inhibitors, other hormonal therapies, acupuncture, meditation, herbal medicines, and mindfulness, the patients decided to undergo diagnostic operative laparoscopy, either with or without robotic assistance, along with hysteroscopy.

**Figure 2:**
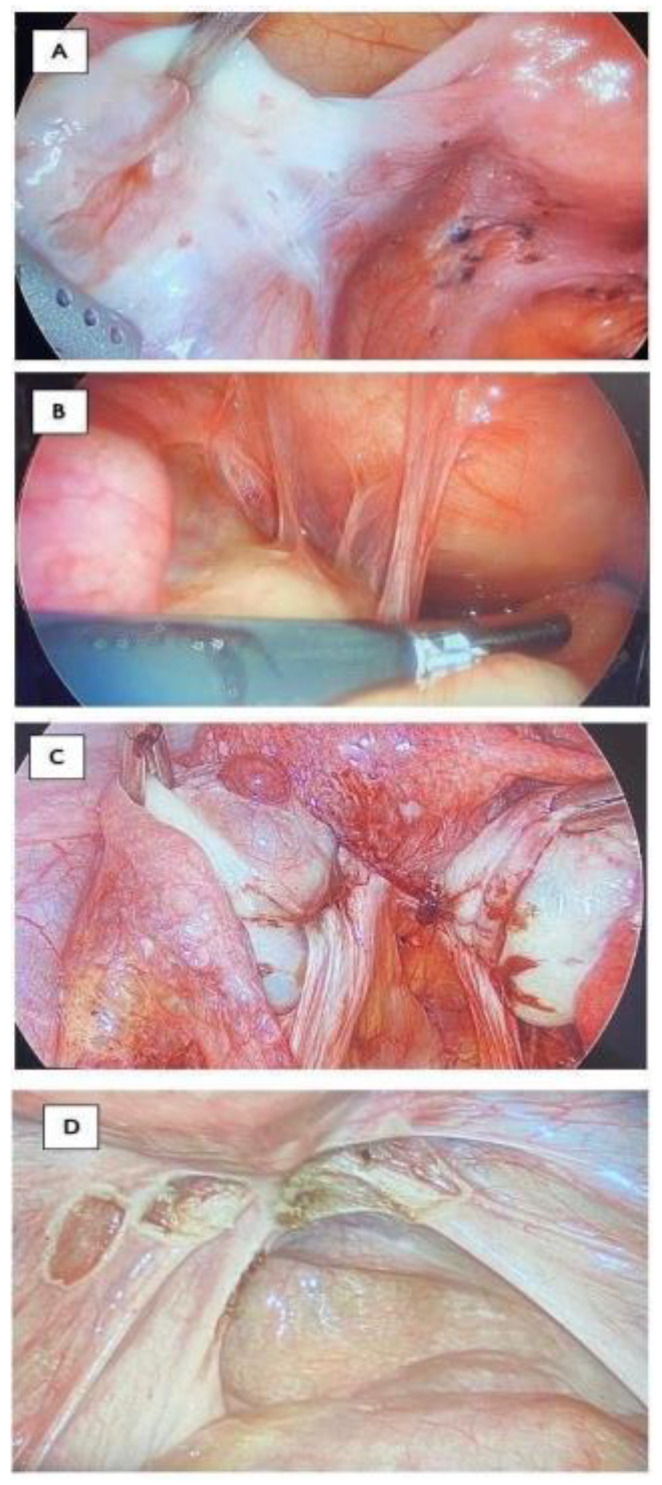
(**A**) Endometriosis of serosa and subserosa of the left ureter in a patient with unexplained infertility, with failed IVF and IUI cycles. (**B**) Filmy peritubal and periovarian adhesions in a patient with normal HSG. (**C**) A case of severe endometriosis with normal HSG and imaging and transvaginal ultrasound. (**D**) Suspicious lesions for endometriosis have been biopsied.

After obtaining informed consent, video-laparoscopy and video-hysteroscopy were performed to assess the abdominal, pelvic, and uterine cavities and to evaluate for tubal patency. Hysteroscopy was completed, and endometrial samplings were collected to assess potential anatomical irregularities within the uterine cavity and to examine endometrial pathologies, including endometritis. Following this procedure, a uterine manipulator was introduced into the uterine cavity. Video-laparoscopy was then performed to evaluate the upper and lower abdominal cavities (see Figure 2) [12]. The Revised American Society for Reproductive Medicine (rASRM) classification system was employed in this study to determine the stage of endometriosis found laparoscopically [62,63].

Following the evaluation, the endometriosis and/or suspicious lesions were excised and sent to the Department of Pathology at Stanford University Medical Center for histological evaluation. This Department of Pathology was chosen for pathology assessment of all patients in this study for their well-established standardized measures and thorough assessments of endometriosis. All patients in this study had their pathology assessment done by one pathology lab within this Department of Pathology as well. The presence and treatment of endometriosis were documented in all operative reports. To ensure consistency and accuracy when reporting results, the pathology reports for each patient were carefully examined and assessed multiple times using a standardized phrase list to recognize and collect any data about the potential presence of endometriosis, including the presence of histological markers suggestive of endometriosis, such as hemosiderin-laden macrophages, mesothelial-lined fibrous tissue, fibrosis, and fibrotic adhesions with chronic inflammation [57,58,59,60,61].

### 2.3. Statistical Analysis

The statistical analyses for this study were conducted using the open-source version of RStudio for Windows, version 4.2.1 (Posit Software, Boston, MA, USA). The patient selection process involved applying our predetermined inclusion and exclusion criteria to select appropriate individuals. The remaining patients formed the basis of our study cohort, which underwent comprehensive statistical analysis. This involved calculating prevalence and proportions across various subgroups through data filtering, tallying, and applying simple mathematical models. Furthermore, the number of patients classified according to each of the four distinct stages of endometriosis was found via programming code, and a fundamental mathematical model was employed to calculate the mean ages, along with their corresponding standard deviations, for each respective variable. Additionally, calculations were manually performed to find specificity, sensitivity, Positive Predictive Value, Negative Predictive Value, and Positive Likelihood Ratio. All calculations were rounded to one decimal place in the final report.

It is important to note that our study had no significant missing data, and some sampling strategies, such as correlation tests, did not apply to this particular study design. To visually present the prevalence and proportions of our data, we created data visualizations using Microsoft Word online software, version 2311 (Microsoft Corporation, Redmond, WA, USA).

## 3. Results

The operative cohort consisted of 215 patients, ranging in age from 25 to 45 years, all of whom met the specified criteria described in the Methods. The mean age of the cohort was 36.2 ± 4.1 years. Out of the patients with a mean age of 36.2 years, 61.9% (*n* = 133) reported previous use of hormonal suppression as a treatment for possible endometriosis in the context of unexplained infertility. Additionally, 83.7% (*n* = 180), with a mean age of 35.8 years, reported undergoing one or more IUI and/or IVF cycles prior to their endometriosis consultation. Some patients had multiple prior modality treatments (see Table 1).

Histological evaluation confirmed the presence of tissue abnormalities in 98.6% (*n* = 212) of the patients, with 90.7% (*n* = 195) showing endometriosis, as defined as the presence of endometrial-like glands and stroma, in their pathology report. Among the remaining 17 patients with tissue abnormalities, 4 exhibited hemosiderin-laden macrophages, and 13 exhibited fibrous tissue and/or mesothelial-lined fibrous tissue with or without adhesions. Three patients had no significant histological findings. Laparoscopic surgery, coupled with histological evaluation, presented a sensitivity of 100.0%, with there being 195 true positive outcomes and 0 false negative outcomes, and a specificity of 0.0%, with there being 0 true negative outcomes and 20 false positive outcomes. A Positive Predictive Value of 90.7%, a Negative Predictive Value of 0.0%, and a Positive Likelihood Ratio of 1.0, were also calculated. Out of the 215 patients, 4 patients had endosalpingiosis, and all of them presented endometrial-like glands and stroma. In addition, out of the 215 patients, 14% (*n* = 30) were found to have plasma cells in the endometrium, a potential indicator of endometritis. Out of these 30 patients, all were found to have tissue abnormalities, with 28 patients having endometrial-like glands and stroma, 1 patient having fibrous tissue, and 1 patient having fibrous tissue with adhesions. The mean age of patients with tissue abnormalities is 36.2 ± 4.1 years (see Table 2).

The clinical staging of macroscopic endometriosis was determined using the rASRM classification system [62,63]. Most patients were diagnosed with either Stage II (mild disease) or Stage IV (severe disease) endometriosis. The breakdown of stages was as follows: Stage I endometriosis was present in 2.4% (*n* = 5 patients), Stage II in 31.6% (*n* = 67 patients), Stage III in 24.5% (*n* = 52 patients), and Stage IV in 41.5% (*n* = 88 patients). The mean age for each stage was calculated as follows: Stage I, 39.2 years; Stage II, 36.1 years; Stage III, 36.5 years; and Stage IV, 36.0 years (see Table 1). 

No major surgical complications were observed among the patients. Eleven individuals experienced minor complications, but all 11 patients fully recovered from their complications, with minimal to mild medical interference in treatment. Minor complications included urinary tract infection, umbilical infection, and bruising on the anterior abdominal wall near the port insertion site. Instances of abdominal bruising resolved naturally with minimal medical interference, while antibiotics were administered to treat urinary tract and skin infections. Additionally, no cases of cancer arising from endometriosis have been reported among the patients in this study.

## 4. Discussion

This study revealed that the majority of patients who encountered unexplained infertility had abnormal pathology. Of these patients, a majority had endometrial-like glands and stroma in their pathology reports. The calculated sensitivity of 100% indicates that laparoscopic surgery, coupled with histological evaluation of excised lesions, is a strong test for capturing all true positive cases and that no diagnoses of endometriosis among the unexplained infertility patients in this study were missed. The calculated specificity of 0%, with 20 false positive outcomes, indicates that laparoscopic surgery may be effective at warranting caution and suspicion for endometriosis, which later can be either confirmed or denied via histological evaluation. The patients in this study displayed a diverse range of disease severity, as determined by the rASRM classification system [62,63]. A significant proportion of patients were diagnosed with mild-to-severe endometriosis (Stages II, III, and IV), indicating a considerable impact of the disease on the reproductive organs. This observation is consistent with the established link between endometriosis and infertility, as higher disease stages typically correspond to greater anatomical involvement and increased challenges in achieving pregnancy.

If left untreated, endometriosis has the potential to significantly reduce fertility rates [64]. Although no randomized control trials (RCTs) exist to ascertain whether surgical treatment leads to enhanced clinical pregnancy rates in individuals diagnosed with severe disease, observational research studies suggest that surgical intervention yields a 30% pregnancy rate for patients with an obliterated cul-de-sac and a 50–60% pregnancy rate for patients following the removal of endometriomas [65]. We have reported excellent pregnancy rates in patients with endometriosis and failed IVF before [48]. Currently, our patients benefit from even better pregnancy rates than what we have reported previously. This could be due to several factors. As our experience has increased over the years, we recognize and treat endometriosis more thoroughly, and assisted reproductive surgery technology and methods have made significant progress in the past many years as well. However, these patients did not get pregnant with those interventions until they underwent surgical intervention.

Surgery may not be the best option for all patients with unexplained infertility seeking desirable fertility outcomes. In these patients, early assessment of patients with unexplained infertility for possible endometriosis may be considered using noninvasive methods. A meta-analysis involving 8984 patients diagnosed with endometriosis revealed that among those with Stage I or II, IVF outcomes indicated reduced rates of fertilization, implantation, and clinical pregnancy [66]. 

Early and accurate diagnosis may be critical, primarily because endometriosis is often a progressive and estrogen-dependent condition that amplifies the susceptibility to certain cancers, notably endometriosis-associated ovarian cancers [67]. Although the pathogenesis of endometriosis remains uncertain, its possible association with ovarian malignancy and the transformation of extra-ovarian endometriosis into malignancy have been confirmed [14,15]. Of note, endosalpingiosis, independent of endometriosis, also increases the association of ovarian cancers [68]. Additionally, numerous individuals experiencing unexplained infertility have undergone fertility drug treatment over extended periods without achieving the desired results. Although not definitive, the existing findings regarding ovarian cancer risk, particularly invasive epithelial carcinoma and non-epithelial neoplasia associated with fertility drug treatment, are substantial. It is worth noting that a stronger link has been identified between the extended utilization of fertility drugs and the occurrence of borderline tumors of the ovaries [69]. Prolonged ovarian hyperstimulation may increase exposure to estrogen levels, which may worsen endometriosis, a condition shown to be associated with a higher risk of cancer [70]. Hence, recognizing endometriosis’ potential for malignancy and the connection between prolonged fertility drug usage and tumor formation underscores the significance of early detection. Additionally, in one cohort study, infertility treatment raised the risk of post-delivery stroke-related hospitalization, noticeable within just 30 days post-delivery [67]. Prioritizing early diagnosis may be crucial to effectively managing this condition. Moreover, diagnosing and addressing endometriosis in patients with a history of unsuccessful IVF may enhance their chances of achieving a successful conception. Pregnancies would also be closely monitored, as it is known that patients with endometriosis have a higher rate of pregnancy complications such as miscarriages, preterm birth, bleeding, and placental abnormalities like placenta previa [13,71]. Lastly, early diagnosis for younger patients may prompt consideration for earlier intervention by specialists, which may potentially prevent disease progression and organ damage, preserve fertility, resolve pain, and provide access to increased surveillance and ongoing individualized care including the possibility of egg freezing if desired and/or indicated [72].

Overall, the high prevalence demonstrated within this group raises the natural question: Are we potentially under-detecting endometriosis? Furthermore, is there a direct link between endometriosis and infertility? As mentioned before, the substantial prevalence observed in our findings highlights the critical importance of early and accurate diagnosis and the necessity for personalized treatment approaches.

It is important to acknowledge the limitations of this study. The study carries significance for individuals already aware of the widespread occurrence of endometriosis and its tendency to be dismissed, resulting in diagnostic and treatment delays. Nevertheless, those holding an alternative viewpoint might perceive the study as leaning towards a bias in favor of endometriosis. Further analysis of the results encourages an open discussion on possible selection biases, intending to further assist patients with unexplained infertility. A specific concern regarding selection bias revolves around whether the notable prevalence can be attributed to patients being intentionally referred to rule out endometriosis or if it is linked to patients under the care of REIs who possess a profound understanding of the connection between unexplained infertility and endometriosis. In a similar vein, the study’s population represents a subcategory within the broader cohort of individuals with “unexplained infertility”. Specifically, it comprises individuals with unexplained infertility who may have experienced unsuccessful prior treatments, alluding to the results indicating that 61.9% of patients reported previous use of hormonal treatments, and 83.7% of patients reported previously undergoing one or more IUI and/or IVF cycles. This selection criterion may introduce a bias into the study’s findings, limiting the generalizability of conclusions to the entire population of individuals with “unexplained infertility”. In addition, the study’s retrospective design introduces inherent limitations, including the possibility of chart reading errors. Other limitations include the absence of details regarding the precise count of unsuccessful IUI and/or IVF cycles undergone by individual patients before seeking assistance for unexplained infertility, the potential destruction of Stage I endometriosis during surgery due to its superficial nature, and the histological evaluation being limited to a fraction of the received tissue. Lastly, it is worth noting that this study does not encompass data regarding pregnancy outcomes following surgical intervention for endometriosis, as they were referred back to their REI referring center.

**Figure 3:**
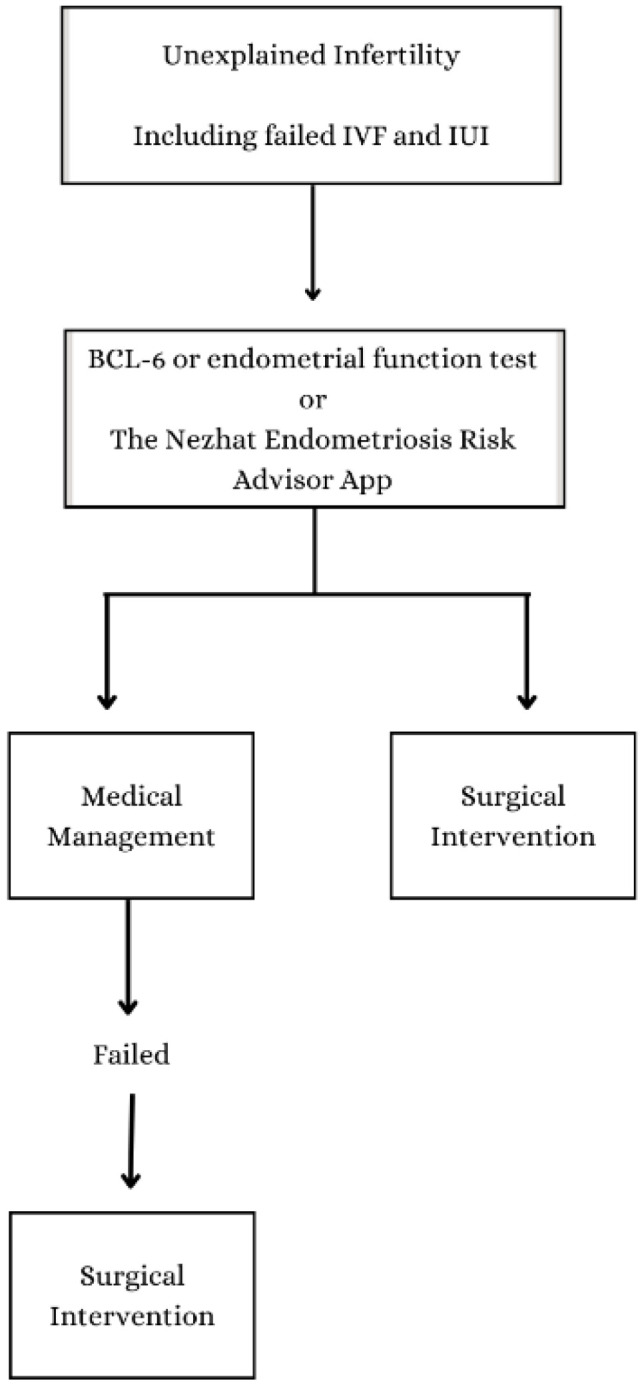
The prevalence of endometriosis in patients with unexplained infertility. This flow chart presents a summary of the diagnosis methodology.

If selection biases have a big effect on the prevalence number we found in our study, the results of this study could encourage infertility centers to do the same kind of research with their own patient groups. Should this study further align with patients undergoing ART treatments for unexplained infertility, it has the potential to significantly enhance our collective efforts in advocating for early diagnosis and treatment for these individuals, helping patients throughout their reproductive lives. The values reported in this study offer distinct viewpoints that can guide future inquiries about the enhancement of endometriosis diagnosis, ultimately contributing to optimal patient care and attaining positive fertility outcomes. Figure 3 provides a flow chart summarizing the diagnosis process and methodology.

## 5. Conclusions

Early diagnosis of endometriosis is essential as it provides crucial insight into the heightened risk of future obstetric and non-obstetric issues in affected patients [11,13,14,15]. The results of this article report that endometriosis is far more common in patients with unexplained infertility than what has been previously reported in the medical literature. Sharing these findings will hopefully bring to light the importance of early diagnosis and will mitigate diagnostic delays, especially since early diagnosis can be helpful to the patients and their physicians in their short- and long-term management. Hence, every attempt should be made for the diagnosis of endometriosis as early as possible. The introduction of dependable, non-invasive, and cost-effective endometriosis markers or screening tools may enhance our diagnostic capabilities, particularly in cases of unexplained infertility [39,40,41,42]. This advancement empowers healthcare providers to comprehensively manage endometriosis patients for both obstetric and non-obstetric conditions.

## Figures and Tables

**Table 1:** Demographic characteristics of the study population.

Variable	Number of Patients (%)	Mean Age ± SD
Mean age of patient cohort	215	36.2 ± 4.1
Endometriosis stage (rASRM) I II III IV	5 (2.4) 67 (31.6) 52 (24.5) 88 (41.5)	39.2 ± 2.8 36.1 ± 4.2 36.5 ± 4.3 36.0 ± 3.9
Had a history of taking hormonal treatments	133 (61.9)	36.2 ± 4.1
Had a history of one or more failed IUI and/or IVF cycles	180 (83.7)	35.8 ± 3.7

**Table 2:** Breakdown of histological analysis conducted during pathology assessment.

Histological Findings	Number of Patients (%)	Mean Age ± SD
Prevalence of histologically-proven tissue abnormalities	212 (98.6)	36.2 ± 4.1
Prevalence of histologically-proven endometriosis	195 (90.7)	36.3 ± 4.0
Presence of endometrial-like glands and stroma	195	36.3 ± 4.0
No presence of endometrial-like glands and stroma, while:		
- significant for the presence of hemosiderin-laden macrophages	4	32.8 ± 5.4
- significant for the presence of fibrous tissue, and/or mesothelial-lined fibrous tissue, and/or adhesions	13	35.8 ± 4.5
- no significant histological findings	3	37.3 ± 6.7
Presence of plasma cells in the endometrium as a possible sign of endometritis	30 (14.0)	34.7 ± 3.5

## Data Availability

Data will be made available to the editors of the journal for review or query upon request.
